# Factors associated with parental hesitancy towards the human papillomavirus vaccine: a cross-sectional study

**DOI:** 10.1038/s41598-025-94067-1

**Published:** 2025-05-26

**Authors:** Monela Mah-Ngum Ntonifor, Nkengeh Ntangatung Tazinkeng, Ben-Lawrence Kemah, Noumbissie Evenge Claudia, Yonta Kenne Sonia, Sangwe Clovis Nchinjoh, Clarence Mvalo Mbanga, Marwa Maweya Abdelbagi Elbasheer, Valirie Ndip Agbor

**Affiliations:** 1 Department of Medical Laboratory Science, Faculty of Health Sciences, University of Buea, 63 Buea, Cameroon; 2 Department of Health Research, Health Education and Research Organization (HERO), 154 Buea, Cameroon; 3 Department of Internal Medicine, Newark Beth Israel Medical Center, Newark, NJ 07112 USA; 4 Ebolowa Regional Hospital Center, 1094 Ebolowa, Cameroon; 5 Division of Public Health, Faculty of Health Sciences, University of the Free State, Bloemfontein, 9300 South Africa; 6 Clinton Health Access Initiative Inc, P.O. Box 2664, Yaoundé, Cameroon; 7 Cancer Epidemiological Unit, Nuffield Department of Population Health, University of Oxford, Oxford, OX3 7LF UK; 8 UK Biobank, Nuffield Department of Population Health, University of Oxford, Big Data Institute Building, Old Road Campus, Oxford, OX3 7LF UK; 9 Global Health Trials Unit, Department of Clinical Sciences, Liverpool School of Tropical Medicine, Pembroke Place, Liverpool, L3 5QA UK

**Keywords:** HPV, Cervical cancer, Parent, Hesitancy, Acceptance, Awareness, Risk factors, Epidemiology, Viral infection

## Abstract

Cervical cancer is the second most common cancer among females in Cameroon, with human papillomavirus (HPV) being the primary cause. While HPV vaccines are highly effective and have been introduced by Cameroon’s Ministry of Health (MOH), uncertainties persist regarding the determinants of HPV-related vaccine hesitancy. This study investigated the factors associated with parental HPV and cervical cancer awareness as well as parental HPV vaccine hesitancy in Cameroon. This cross-sectional study included 1,187 participants residing in Buea Health District (BHD) from August 2023 to March 2024. A pretested questionnaire adapted from the WHO’s vaccine hesitancy tool was used for data collection. Multivariable logistic regression generated adjusted odds ratios for lack of awareness and vaccine hesitancy. Younger ages and lower education levels were significantly associated with greater odds of HPV and cervical cancer unawareness. Parents with lower educational attainment and those unaware of HPV and cervical cancer had higher odds of vaccine hesitancy. Additionally, concerns about vaccine safety and side effects, distrust in the MOH and pharmaceutical companies, and lack of support from religious leaders were associated with parental vaccine hesitancy. In contrast, parents with a history of chronic illness had lower odds of being hesitant. The study identified several factors associated with parental HPV vaccine hesitancy. Addressing these factors could play a key role in improving vaccine uptake among children and eventually reducing cervical cancer rates in Cameroon.

## Introduction

Human Papillomaviruses (HPVs) are potentially oncogenic viruses that account for over 90% of cervical cancer cases^1^. HPVs also cause other cancers, including cancers of the oropharynx, anus, vagina, penis, and vulva^1^. Transmission of these viruses most commonly occurs through sexual intercourse, however, several reports have documented vertical transmission from mother to child^1^. Despite being preventable and curable, cervical cancer is the fourth most diagnosed cancer among females worldwide^2^. The prevalence of cervical cancer is highest in sub-Saharan Africa, where awareness and access to population-based prevention services are limited^3,4^. In Cameroon, cervical cancer is the second most frequent cancer among females aged 15–44 years^5^, with an incidence rate of 14.9%, accounting for 14.8% of cancer-related deaths^6^. Annually, about 2,770 new cervical cancer cases are diagnosed in Cameroon with about 1,787 of them dying each year^7^.

In 2018, the World Health Organisation (WHO) earmarked cervical cancer for elimination by 2030 due to its significant global health burden^8^. Complete vaccination of at least 90% of girls against HPV by the age of 15 years is a chief goal to achieve cervical cancer elimination^9^. High-income countries with relatively high HPV vaccination coverages have seen significantly lower rates of cervical cancer, highlighting the vaccine’s effectiveness in cervical cancer prevention^10^. However, HPV vaccination programs in most African countries, including Cameroon, are hampered by inadequate uptake of HPV vaccines, lack of funding, and substandard monitoring^11^. A meta-analysis estimated the uptake rate of HPV vaccination among adolescents in sub-Saharan Africa at 28.5%^12^; the rate was highest in Kenya (62.5%) and lowest in Nigeria (3.8%). However, according to reports from the International Agency for Research on Cancer (IARC), uptake of the vaccine among eligible girls in Cameroon in 2020 was only 5%^7^.

The HPV vaccine was incorporated into the Expanded Program on Immunisation (EPI) schedule in late 2020 for 9-year-old girls, following a two-dose schedule. In early 2023, the Ministry of Health in Cameroon, in accordance with WHO’s recommendations, implemented a single-dose vaccination schedule to improve vaccine coverage and curb viral transmission^7,13^. The single-dose HPV vaccine regimen has been demonstrated to be non-inferior to the two-dose regimen^13–15^. In addition, a single dose vaccine is expected to improve vaccine coverage by reducing costs and logistic challenges associated with the delivery of the two-dose HPV vaccine regimen^13^.

HPV vaccination is recommended for adolescents before their first sexual exposure to prevent cervical cancer^16^. However, vaccine hesitancy, particularly parents hesitancy towards vaccination of their children (parental hesitancy), is a major limitation to HPV and cervical cancer elimination programs^17,18^. Vaccine hesitancy is a delay in acceptance or refusal of vaccines despite their availability and often influenced by complex and context-specific factors^19^. Since the onset of the coronavirus disease 2019 (COVID-19) pandemic, conspiracy theories against vaccines have further exacerbated HPV vaccine hesitancy^18^. This study investigated the factors associated with (1) parental HPV and cervical cancer awareness and (2) parental HPV vaccine hesitancy.

## Methods

### Study design and setting

This community-based cross-sectional study was conducted in the Buea Health District (BHD) from August 2023 to March 2024. Buea, the regional capital of the South West Region of Cameroon, has an estimated population of 177,297 inhabitants and is the home to the largest English-speaking university in the country, the University of Buea. The BHD covers a total surface area of 870 km^2^ and consists of seven health areas: Bokwango, Bova, Buea Road, Buea Town, Molyko, Muea, and Tole.

### Participants

We included parents of children aged 9–18 years who were residents of the BHD and consented to participate in the study. We excluded individuals visiting the BHD and those who withdrew consent during the study.

### Sample size and sampling

We estimated that a minimum of 943 participants would be needed to detect an odds ratio (OR) of 1.5 with a power of 80% and a two-sided alpha of 0.05. The proportion (0.31) of parental HPV hesitancy was obtained from a study conducted in Northwest Ethiopia^20^.

We used a two-stage sampling method to recruit participants. In the first stage, we randomly selected four of the seven health areas in the BHD: Bokwango, Bueatown, Molyko, and Muea. In the second stage, parents were recruited by convenient sampling from community centres (e.g., marketplaces, schools, hospitals, meeting houses, places of worship) and their homes. Participants were selected using convenience sampling because it requires significantly fewer financial and human resources compared to random sampling^21^. The number of participants recruited per health area was allocated proportionally to the population size of each health area, as estimated by the South West Regional Delegation of Public Health.

### Data collection

Five individuals were selected from the study areas and trained in interviewing participants and data recording to minimise the risk of social desirability and interviewer bias during data collection. Data were collected using a modified version of the WHO’s standardised questionnaire (version 2.0) designed to assess COVID-19 vaccine hesitancy in adults^22^. The COVID-19 questionnaire consisted of seven sections: sociodemographic factors, medical history, history of vaccination, perceived risk of COVID-19, confidence in the vaccine, willingness to receive vaccine, and social influences on vaccination decisions. The questionnaire for the present study retained most items from the original WHO questionnaire but excluded questions not relevant to the present study, including perceived stigma related to the participants getting HPV and freedom of participant to meet family and friends without infecting them.

The questionnaire for the following study was structured as follows:

- Sociodemographic factors: age, gender, marital status (single, married, cohabiting, divorced, widow/widower), highest level of education, religion, employment status, monthly household income (in FCFA), and number of children.
- Medical history: History of chronic illness (yes or no).
- History of vaccination: Receipt of recommended and other vaccines for children (yes or no) and parental vaccination (yes or no).
- HPV-related awareness: Awareness of HPV (yes or no), HPV vaccine (yes, no, or not sure), cervical cancer (yes or no), or HPV as a cause of cervical cancer (yes or no).
- Perceived risk of cervical cancer: Perceived risk of cervical cancer as serious disease (yes or no).
- Perceived vaccine safety: Perceived safety of HPV vaccine for children (safe, not safe, or not sure) and concerns about severe vaccine adverse effects (yes, no, or not sure).
- Social influence: Participants think their religious leaders will support children receiving the HPV vaccine (yes, no, not sure); participants have heard any bad news regarding the HPV vaccine (yes or no).
- Trust: Trust in pharmaceutical companies manufacturing vaccines (yes, no, or somewhat) and trust in the MOH (yes, no, or somewhat).
- Willingness to vaccinate child: Participants were asked whether their children had received the HPV vaccine (yes, no, or not sure) and, if not, whether they intended to vaccinate them in the future (yes, no, or not sure).

The adapted questionnaire was then pretested among 20 randomly selected parents living in the Tole health area who were not included in the study. The questionnaire was administered via interviews in English and took 15 min on average to complete.

### Outcomes and definitions

The primary outcome of this study was parental HPV vaccine hesitancy, defined as parents’ unwillingness to accept the HPV vaccine for their female children. Parental HPV vaccine hesitancy was assessed using the question, “Has your child taken the HPV vaccine?” and the responses were “Yes,” “No,” and “Not sure.” Participants who responded “No” or “Not sure” to the previous question were asked a follow-up question: “Do you intend to give them the vaccine?”; a “No” or “Not sure” response depicted HPV vaccine hesitancy.

The secondary outcomes were HPV infection and cervical cancer awareness. Cervical cancer awareness was assessed by asking, “Have you heard of cervical cancer?” and HPV awareness was assessed using the question “Have you heard of HPV?”. The responses were either “Yes” or “No.” Participants who responded “No” were considered unaware.

### Ethical approval and considerations

Ethical approvalwas obtained from the Institutional Review Board of the University of Buea (2023/1949-02), followed by administrative approval from the South West Regional Delegation of Public Health. This study was conducted in accordance with the principles of the Declaration of Helsinki. Participation in the survey was voluntary, and participants’ autonomy and anonymity were assured. All participants provided informed consent before enrolment into this study.

### Statistical analysis

All analyses were performed using R programming software (version 4.3.1, 2023, The R Foundation for Statistical Computing, Vienna, Austria). Categorical variables were summarised using frequency and percentage, while quantitative variables were summarised using mean and standard deviation (SD).

Multivariable logistic regression generated adjusted ORs and 95% confidence intervals (CIs) for factors associated with lack of awareness of HPV infection and cervical cancer after adjusting for age, age-squared, sex, health area, marital status, education, employment status, monthly household income, and history of chronic disease (objective 1). To investigate the factors associated with parents’ hesitancy towards HPV vaccination for their children (objective 2), we additionally adjusted for the number of children and the number of children squared as covariates.

The responses of participants recruited from one health area might be more similar than those of participants from other health areas, introducing clustering into the data. Clustering underestimates standard errors from models such as generalised linear models and inflates type I error rates, although it does not affect estimates of the regression coefficients^23^. To account for clustering by health area, the “vcovCL” function from the *sandwish* package was used for clustered covariance estimation and the “coeftest” function from the *lmtest* package was used to generate robust standard errors and p-values.

For analyses of ordered categorical variables (like level of education), the Floating Absolute Risk (FAR) method was used to calculate the variance of the log OR for each category, including the reference category^23^. This group-specific variance reflects the amount of information in that category and was used to calculate the group-specific CI. This method enables pairwise comparisons of the odds of an outcome variable (e.g., vaccine hesitancy) across any two categories rather than restricting comparisons between other categories to an arbitrarily defined reference category^23^. The group-specific FARs and their 95% CIs were then plotted to visualise the shape of associations. The group-specific 95% CIs are narrower than classical CIs and were, therefore, only used for plotting; robust CIs were used when reporting the odds in one group compared to the reference category^23^.

For analysis of the lack of awareness of HPV and cervical cancer, two-tailed p-values less than 0.05 were considered statistically significant. For analysis of vaccine hesitancy, two-tailed p-values were corrected using Bonferroni’s method by dividing the conventional threshold (0.05) by the number of exposures investigated (0.05/19 = 0.0026). Therefore, two-tailed p-values less than the Bonferroni threshold of 0.0026 were considered statistically significant. There were no missing data in the dataset used for this study.

## Results

### Characteristics of the study population

This study included 1,187 participants with a mean age of 42.4 years (SD = 11.5, range = 20–87).

Most of the participants were aged 30–39 years (34.4%), females (66.5%), married (57.9%), had non-manual employment (48.5%), and had attained a tertiary level of education (29.7%) (Table 1). Approximately a third of the participants were from the Molyko health area (36.4%), whereas 17.3% were from Buea Town.

**Table 1:** Characteristics of study participants.

Characteristics^§^	ALL (*N* = 1187)
Age, year	
20–29	136 (11.5)
30–39	408 (34.4)
40–49	357 (30.1)
50–59	181 (15.2)
60+	105 (8.85)
Age, mean (SD), year	42.4 (11.5)
Health area	
Bokwango	261 (22.0)
Buea town	205 (17.3)
Molyko	432 (36.4)
Muea	289 (24.3)
Sex	
Female	789 (66.5)
Male	398 (33.5)
Education	
No formal schooling	64 (5.39)
Primary	210 (17.7)
Secondary	270 (22.7)
Post-secondary	291 (24.5)
Tertiary	352 (29.7)
Marital status	
Single	281 (23.7)
Married	687 (57.9)
Cohabitation	72 (6.07)
Divorced	29 (2.44)
Widow/Widower	118 (9.94)
Employment status	
Employed (manual)	348 (29.3)
Employed (non-manual)	576 (48.5)
Unemployed	263 (22.2)
Monthly household income, in 10^3^ FCFA (in USD)	
<50 (< 83.24)	241 (20.3)
50 – <100 (83.24 – <166.48)	392 (33.0)
100 – <150 (166.48 – <249.72)	237 (20.0)
150 – <200 (249.72 – <332.96)	126 (10.6)
≥ 200 (≥ 332.96)	191 (16.1)
Religion	
Pentecostal	372 (31.3)
Catholic	282 (23.8)
Presbyterian	262 (22.1)
Baptist	161 (13.6)
Islam	39 (3.29)
None	71 (5.98)
Number of children, mean (SD)	3.71 (2.02)

*SD* standard deviation. ^§^Data is presented as frequency (percentage) unless stated otherwise. Cohabitation: state of living together and having a sexual relationship without being married.

### Factors associated with HPV and cervical cancer unawareness

**Fig. 1:**
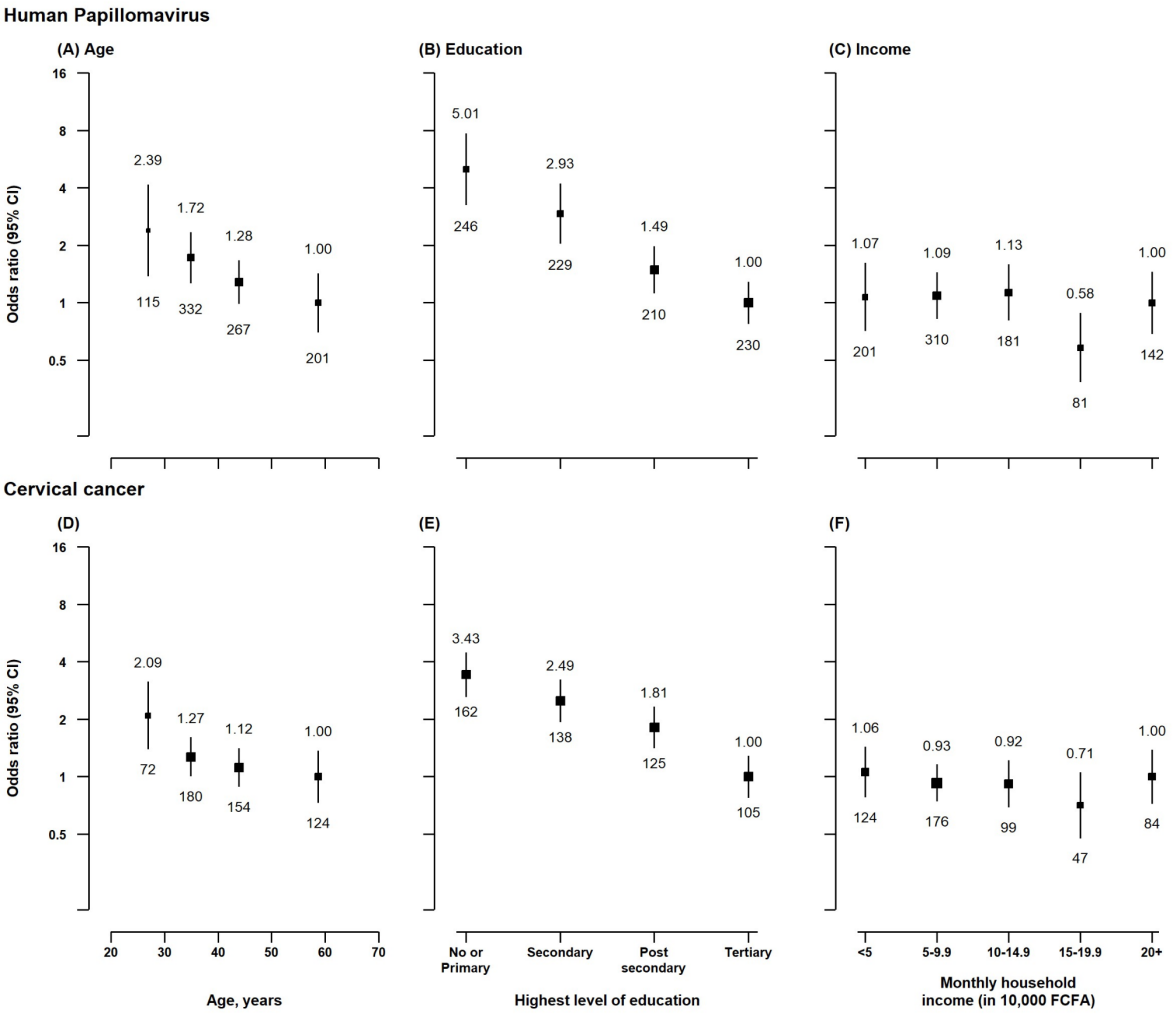
Associations of age, education, and income with lack of awareness of human papillomavirus and cervical cancer. Separate models were fitted for each exposure and adjusted for age, age-squared, sex, health area, marital status, education, employment status, monthly household income, and history of chronic disease. The black squares are adjusted odds ratios weighted by the inverse of the variance, which represents the amount of information in each exposure category. The vertical lines represent the 95% floating absolute risk confidence intervals. The numbers on the vertical lines are the adjusted odds ratio, while the numbers below are the number of participants unaware of human papillomavirus or cervical cancer in each exposure group.

A total of 272 (22.9%) and 657 (55.3%) of the 1,187 participants reported being aware of HPV infection and cervical cancer, respectively. Figure 1 shows the factors associated with HPV and cervical cancer unawareness. Younger age and lower educational attainment were significantly associated with higher odds of HPV and cervical cancer unawareness (Fig. 1; Table 2). Compared to participants aged ≥ 50 years, those 20–29 years had a 2.3-fold higher odds of HPV unawareness (OR: 2.39; CI: 1.30–4.27; *p* = 0.0030) and a 2.1-fold higher odds of cervical cancer unawareness (2.09; 1.31–3.34; *p* = 0.0019). Additionally, participants with no formal education or those who had at most primary education had 5.0-fold greater odds of HPV unawareness (5.01; 4.07–6.17; *p* < 0.0001) and 3.4-fold higher odds of cervical cancer unawareness (3.43; 3.09–3.82; *p* < 0.0001) compared to those with tertiary education. In contrast, participants who reported being divorced or widowed had 56% lower odds of HPV unawareness compared to those who reported being single (0.44; 0.26–0.76; *p* = 0.0028).

**Table 2:** Factors associated with lack of awareness of HPV and cervical cancer.

Exposure	HPV				Cervical cancer			
	*n*	*N*	OR (95 CI) ^†^	*P*-value^†^	*n*	*N*	OR (95 CI)^†^	*P*-value^†^
Age, years								
50+	201	286	Reference		124	286	Reference	
40–49	267	357	1.28 (1.08–1.53)	**0.0056**	154	357	1.12 (0.93–1.34)	0.2289
30–39	332	408	1.72 (1.30–2.28)	**0.0001**	180	408	1.27 (0.99–1.63)	0.0619
20–29	115	136	2.39 (1.34–4.27)	**0.0030**	72	136	2.09 (1.31–3.34)	**0.0019**
Sex								
Female	641	789	Reference		345	789	Reference	
Male	274	398	0.67 (0.38–1.17)	0.1571	185	398	1.34 (0.82–2.19)	0.2395
Marital status								
Single	223	281	Reference		133	281	Reference	
Married	550	687	1.01 (0.79–1.28)	0.9608	300	687	0.91 (0.54–1.51)	0.7046
Cohabiting	49	72	0.57 (0.19–1.66)	0.3002	30	72	0.77 (0.33–1.78)	0.5384
Divorced/widow/widower	93	147	0.44 (0.26–0.76)	**0.0028**	67	147	1.05 (0.44–2.50)	0.9178
Education								
Tertiary	230	352	Reference		105	352	Reference	
Post-secondary	210	291	1.49 (1.27–1.74)	**< 0.0001**	125	291	1.81 (1.38–2.38)	**0.0001**
Secondary	229	270	2.93 (2.14–4.03)	**< 0.0001**	138	270	2.49 (1.73–3.59)	**< 0.001**
No formal or primary	246	274	5.01 (4.07–6.17)	**< 0.0001**	162	274	3.43 (3.09–3.82)	**< 0.001**
Employment status								
Unemployed	211	263	Reference		125	263	Reference	
Employed (manual)	260	348	0.70 (0.34–1.47)	0.3497	156	348	0.91 (0.55–1.49)	0.6974
Employed (non-manual)	444	576	0.89 (0.47–1.70)	0.7259	249	576	0.98 (0.69–1.38)	0.9033
Monthly household income								
200+	142	191	Reference		84	191	Reference	
150–<200	81	126	0.58 (0.22–1.50)	0.2632	47	126	0.71 (0.57–0.88)	**0.0015**
100–<150	181	237	1.13 (0.68–1.88)	0.6328	99	237	0.92 (0.76–1.11)	0.3636
50–<100	310	392	1.09 (0.64–1.84)	0.7474	176	392	0.93 (0.63–1.36)	0.6943
< 50	201	241	1.07 (0.54–2.13)	0.8423	124	241	1.06 (0.84–1.32)	0.6341
Chronic disease								
No	697	903	Reference		413	903	Reference	
Yes	218	284	1.12 (0.82–1.52)	0.4908	117	284	0.86 (0.56–1.33)	0.5095
Parent has received any vaccination								
Yes	734	958	Reference		409	958	Reference	
No	181	229	1.23 (0.75–2.02)	0.4166	121	229	1.36 (0.92–2.01)	0.1287

Separate models were fitted for each exposure, with adjustments for age, age-squared, sex, health area, religion, marital status, education, employment status, monthly household income, history of chronic disease, number of children, and number of children-squared. Significant p-values are written in bold. n = Number of participants who were unaware, N = Total participants per group. ^**†**^The 95% confidence interval (CI) and p-values were estimated using robust standard errors with health areas as clusters.

There was no significant association between sex, employment status, chronic disease, and a history of parents’ vaccination with HPV and cervical cancer unawareness. Additionally, marital status was not associated with cervical cancer unawareness.

### Factors associated with parental HPV vaccine hesitancy

A total of 555 (46.8%) of the 1,187 participants were hesitant about the HPV vaccine. Multivariable logistic regression analysis identified education and knowledge of HPV and cervical cancer to be significantly associated with HPV vaccine hesitancy. Parents with no formal education or only primary education (1.31; 1.12–1.53; *p* = 0.0008) and secondary education (1.27; 1.09–1.49; *p* = 0.0023) were more likely to be hesitant to HPV vaccination compared to parents with tertiary education (Table 3). Participants unaware of HPV (3.09; 2.09–4.58; *p* < 0.0001) or cervical cancer (1.93; 1.55–2.39; *p* < 0.0001) had higher odds of HPV vaccine hesitancy compared to those who were aware. Furthermore, parents who did not perceive cervical cancer as a serious condition (1.93; 1.20–3.09; *p* = 0.0076) or were unsure about its severity (2.43; 2.01–2.95; *p* < 0.0001) were more likely to be hesitant (Table 3).

**Table 3:** Factors associated with parental HPV vaccine hesitancy.

Characteristics	*n*	*N*	OR (95% CI)	*P*-value^§^
Age, years (ref: 50+)				
40–49	164	357	0.79 (0.57–1.10)	0.1637
30–39	190	408	0.84 (0.71–1.01)	0.0618
20–29	56	136	0.73 (0.52–1.02)	0.0653
Female (ref: male)	186	398	0.88 (0.57–1.38)	0.5867
Marital status (ref: single)				
Married	319	687	0.92 (0.72–1.17)	0.4796
Cohabiting	38	72	1.20 (0.76–1.89)	0.4362
Divorced/widow/widower	70	147	0.84 (0.58–1.20)	0.3276
Education (ref: tertiary)				
Post-secondary	133	291	1.10 (0.98–1.24)	0.1166
Secondary	134	270	1.27 (1.09–1.49)	**0.0023**
No formal or primary	136	274	1.31 (1.12–1.53)	**0.0008**
Employment status (ref: unemployed)				
Employed (manual)	165	348	1.01 (0.73–1.40)	0.9408
Employed (nonmanual)	267	576	0.97 (0.69–1.37)	0.8803
Monthly household income, in 10^3^ FCFA (Ref: 200+)				
200+	93	191	Reference	
150 – <200	65	126	1.08 (0.45–2.58)	0.8636
100 – <150	111	237	0.92 (0.67–1.27)	0.6191
50 – <100	182	392	0.93 (0.53–1.63)	0.8132
< 50	104	241	0.84 (0.40–1.77)	0.6514
Parents chronic disease (yes: ref: no)	106	284	0.58 (0.51–0.67)	**< 0.0001**
Parent has received any vaccination (no: ref: yes)	112	229	1.06 (0.88–1.28)	0.5537
Child has received a recommended vaccine (ref: yes)				
No	17	30	1.38 (1.06–1.80)	0.0152
Not sure	23	50	0.89 (0.67–1.17)	0.3878
Heard of HPV (no: ref: yes)	477	915	3.09 (2.09–4.58)	**< 0.0001**
Heard of cervical cancer (no: ref: yes)	295	530	1.93 (1.55–2.39)	**< 0.0001**
Thinks cervical cancer a serious disease (ref: yes)				
No	19	36	1.93 (1.20–3.09)	**0.0067**
Not sure	36	61	2.43 (2.01–2.95)	**< 0.0001**
Never heard of cervical cancer	295	530	2.19 (1.71–2.80)	**< 0.0001**
Child has received other vaccines (ref: yes)				
No	20	36	1.33 (0.92–1.94)	0.1288
Not sure	24	52	0.88 (0.51–1.52)	0.6497
Thinks vaccine is safe (ref: safe)				
Not safe	127	165	9.70 (7.57–12.42)	**< 0.0001**
Not sure	272	452	4.12 (2.96–5.75)	**< 0.0001**
Concerns about adverse effects (ref: no)				
Not sure	211	417	3.18 (2.28–4.42)	**< 0.0001**
Yes	287	541	3.75 (3.19–4.41)	**< 0.0001**
Religious leader supports vaccination (ref: yes)				
No	102	162	3.40 (2.68–4.31)	**< 0.0001**
Not sure	286	533	2.29 (2.05–2.56)	**< 0.0001**
Trust in the MOH (ref: yes)				
No	210	310	4.09 (3.02–5.53)	**< 0.0001**
Somewhat	146	294	1.92 (1.52–2.43)	**< 0.001**
Trust in pharmaceutical companies (ref: yes)				
No	247	387	3.66 (2.75–4.87)	**< 0.0001**
Somewhat	155	337	1.75 (1.31–2.35)	**0.0002**
Heard bad news about vaccine (yes: ref: no)	4	12	0.57 (0.07–4.38)	0.5901

Separate models were fitted for each exposure, with adjustments for age, age-squared, sex, health area, religion, marital status, education, employment status, monthly household income, history of chronic disease, number of children, and number of children-squared. *HPV* human papillomavirus, *n* number of participants who were hesitant, *N* total participants per group, *OR* odds ratio, *MOH* ministry of health. ^**§**^The 95% confidence interval (CI) and p-values were estimated using robust standard errors with health areas as clusters. Significant values are in bold.

Participants who believed the vaccine was unsafe were nine times more likely to be hesitant than participants who perceived it to be safe (9.70; 7.57–12.42; *p* < 0.0001). Concerns about potential adverse effects of the vaccine were also associated with a 3.7-fold higher odds of hesitancy (3.75; 3.91–4.41; *p* < 0.0001). Moreover, participants whose religious leaders would not support HPV vaccination were 3.4 times more likely to be hesitant (3.40; 2.68–4.31; *p* < 0.0001).

Distrust in the Ministry of Health (MOH) or pharmaceutical companies was also associated with vaccine hesitancy. Participants who lacked trust in the MOH had four times higher odds of hesitancy (4.09; 3.02–5.53; *p* < 0.0001), and those distrusting pharmaceutical companies were 3.7 times more likely to be hesitant (3.66; 2.75–4.87; *p* < 0.0001).

In contrast, parents with a history of chronic disease were less likely to be hesitant (0.58; 0.51–0.67; *p* < 0.0001).

## Discussion

This study investigated the factors associated with HPV infection and cervical cancer awareness and parental HPV vaccine hesitancy in the BHD. Younger ages and lower educational attainment were associated with HPV and cervical cancer unawareness. Additionally, several factors were significantly associated with parental vaccine hesitancy, including lower educational attainment, a history of chronic disease among parents, lack of awareness of HPV or cervical cancer, concerns about vaccine safety and side effects, from having religious leaders who do not support vaccination, and distrust in the MOH or pharmaceutical companies.

In this study, parents with less than secondary were more likely to be hesitant, and this is consistent with the findings of a study on COVID-19 vaccine hesitancy^24^. Individuals with lower levels of education have limited access to health information, predisposing them to misinformation and misconceptions about vaccination^25,26^. The present study showed that lack of HPV and cervical cancer awareness and not perceiving cervical cancer as a serious disease were associated with higher odds of parental vaccine hesitancy. Further analysis indicated that lower educational attainment was associated with the lack of HPV and cervical cancer awareness, suggesting that educational attainment might influence HPV vaccine hesitancy through HPV and cervical cancer awareness. In contrast, people with higher levels of education have better access to credible sources of health information and better critical thinking skills to make informed decisions about vaccination and are able to decipher messages worth considering from those that are scientifically unsound^27^. Greater educational attainment has been associated with higher vaccination rates^28^.

In line with previous studies from East Africa, concerns about vaccine safety and potential side effects were significantly associated with parental vaccine hesitancy^29,30^. Similar concerns have been reported in studies on other vaccines, including malaria and COVID-19 vaccines^31–33^. Such concerns are exacerbated by misinformation and conspiracy theories about vaccines^31,34^. The present study had limited statistical power to investigate the association between negative information about the vaccine and hesitancy, as only 16 (1.3%) participants admitted hearing bad news about the HPV vaccine. Health education from trained professionals is critical to address misinformation and alleviate vaccine-related concerns^35^. Transparent communication about vaccine side effects can build trust and strengthen community confidence in vaccine programs^27^. Moreover, promoting the proven high efficacy and safety of HPV vaccines can further reassure parents and improve vaccine uptake for their children^24,27^.

Parents with chronic illnesses were less likely to be hesitant. Studies on COVID-19 vaccination found that individuals with chronic health conditions are more likely to get vaccinated owing to their heightened vulnerability^33,36^. Individuals with chronic illness are more likely to be aware of the risks associated with infections and appreciate the relevance of vaccines in mitigating these risks. Hence, these parents are more likely to be proactive in vaccinating their children.

Participants whose religious leaders did not support vaccination were more likely to be hesitant. Studies have shown that young girls whose religion prohibits vaccination against sexually transmissible infections like HPV are more likely to be hesitant^37^. Religion is a well-established determinant of vaccine hesitancy, which could be explained by religious leaders relying on faith-based reasoning rather than medical recommendations. Some religious groups can view disease outcomes to be controlled by divine will or as a test of faith, discouraging the use of vaccines for disease prevention^38^. In addition, distrust in medical authorities by religious communities can fuel vaccine hesitancy, especially when vaccine promotion is perceived as conflicting with their religious or ethical views^38^. For instance, some religious leaders believe that vaccinating adolescents against sexually transmitted diseases can promote promiscuity^37^.

A previous study in Cameroon reported that parents expressed fear and lack of trust in the source of routine vaccines^39^. The present study showed that distrust in the MOH or pharmaceutical companies was significantly associated with HPV vaccine hesitancy among parents. Similar findings have shown that distrust in health authorities or pharmaceutical companies was associated with higher risks of COVID-19 vaccine hesitancy^31,33,40^ with a report from the WHO working group on vaccine hesitancy listing distrust as a chief determinant of vaccine hesitancy^41^.

The findings of this study underscore the importance of addressing factors associated with HPV vaccine hesitancy as a key strategy for preventing cervical cancer. Given the inverse association of educational attainment and awareness of HPV infection and cervical cancer with vaccine hesitancy, the MOH should prioritise targeted and culturally sensitive educational programs^40,42^. Using trusted community staff or community-based organisations for sensitisation campaigns and sharing information on vaccines can be essential in overcoming HPV vaccine hesitancy, ensuring high vaccination rates, and improving the public’s knowledge of HPV and cervical cancer^24^. Involving community leaders, religious figures, and local healthcare providers can also help build trust and encourage vaccine acceptance in the community^43^. These influential figures can bridge the gap between public health authorities and the community and address vaccine-related concerns in a culturally sensitive manner. Furthermore, providing transparent and up-to-date information about vaccine safety and efficacy can help build trust between the MOH and the local community and address the trust issues and concerns about vaccine safety^42^.

This study has some limitations. First, it included only one health district and might not be generalisable to other health districts in Cameroon. Second, there is the possibility of selection bias as parents were recruited mostly at their homes, public places, and social houses, and the study participants might be different from the rest of the population. In addition, although efforts were made to account for important confounders, unmeasured and residual confounding cannot be ruled out. Larger studies using random sampling techniques to select participants are necessary to confirm the results reported in this study.

In conclusion, the findings of this study highlight several key factors that are associated with parental vaccine hesitancy in the BHD, including lower educational attainment, limited awareness of HPV and cervical cancer, concerns related to vaccine safety and side effects, lack of support from religious leaders, and distrust in the MOH and pharmaceutical companies. Public health authorities should focus on designing targeted and culturally sensitive interventions and involve relevant stakeholders to improve HPV vaccine uptake as part of the cervical cancer prevention plan.

## Data Availability

Data that support the findings of this study are available from the corresponding author upon reasonable request.
